# DNASynth: A Computer Program for Assembly of Artificial Gene Parts in Decreasing Temperature

**DOI:** 10.1155/2015/413262

**Published:** 2015-01-06

**Authors:** Robert M. Nowak, Anna Wojtowicz-Krawiec, Andrzej Plucienniczak

**Affiliations:** ^1^Institute of Electronic Systems, Warsaw University of Technology, Nowowiejska 15/19, 00-665 Warsaw, Poland; ^2^Institute of Biotechnology and Antibiotics, Staroscinska 5, 02-512 Warsaw, Poland

## Abstract

Artificial gene synthesis requires consideration of nucleotide sequence development as well as long DNA molecule assembly protocols. The nucleotide sequence of the molecule must meet many conditions including particular preferences of the host organism for certain codons, avoidance of specific regulatory subsequences, and a lack of secondary structures that inhibit expression. The chemical synthesis of DNA molecule has limitations in terms of strand length; thus, the creation of artificial genes requires the assembly of long DNA molecules from shorter fragments. In the approach presented, the algorithm and the computer program address both tasks: developing the optimal nucleotide sequence to encode a given peptide for a given host organism and determining the long DNA assembly protocol. These tasks are closely connected; a change in codon usage may lead to changes in the optimal assembly protocol, and the lack of a simple assembly protocol may be addressed by changing the nucleotide sequence. The computer program presented in this study was tested with real data from an experiment in a wet biological laboratory to synthesize a peptide. The benefit of the presented algorithm and its application is the shorter time, compared to polymerase cycling assembly, needed to produce a ready synthetic gene.

## 1. Introduction

An artificial gene is the DNA strand of a given sequence, synthesized* in vitro*.* De novo* synthesized DNA molecules, without needing an initial DNA template, have higher cost efficiency and higher flexibility than redesigning molecules produced by molecular cloning [1].

The chemical synthesis method for DNA strand uses the repeated addition of nucleotides [2]. It has practical limitations of strand length to about 80 nucleotides (nt) [3]. The creation of artificial genes, usually with length greater than 300 nt, requires the assembly of long DNA molecules from shorter chemically synthesized fragments. The assembly methods typically mix the fragments together in one tube. Thus, successful synthesis requires removing regions having secondary structures with high energy from the fragments, such as repetitive structures, inverted repeats, and regions of extraordinary high or low GC content. If there is no possibility of meeting these conditions, the fragments of a particular gene can only be synthesized by splitting the procedure into several consecutive steps and a final assembly of shorter subsequences, which, in turn, leads to a significant increase in time and labor needed for its production.

Polymerase cycling assembly (PCA) is the most widely used technique [4, 5] to produce long (e.g., 1000 nt) DNA strands from shorter fragments. This method uses the same reactions and reagents as the polymerase chain reaction (PCR). Chemically synthesized shorter fragments are mixed together in one tube and then polymerase builds the complementary strand using the fragments as primers. PCR-specific starters are added and finally the long DNA molecule is amplified. PCA requires the melting temperatures of the overlapping regions to be similar for all fragments. The necessary primer optimization should be performed using specialized oligonucleotide design programs; several solutions for automated primer design for gene synthesis using PCA have been reported [6]. The correct syntheses of an 800 nt strand have also been reported [7].

In the present procedure we do not use PCA, because the long molecule synthesis using PCA requires several steps, where each step extends the output molecule by hybridization, polymerization, and denaturation. We extend the assembly method using ligase chain reaction (LCR) [8, 9]. This method uses exactly one sequence of denaturation, hybridization, polymerization, and ligation. The chemically synthesized DNA strands are mixed together; then the* T4 ligase* enzyme connects the fragments. Next, polymerase creates missing pieces and finally PCR is used to amplify the output molecule. The proposed modification of the assembly method by ligation is carrying out the reaction at decreased temperatures, which helps in providing deterministic assembly of fragments.

The artificial gene synthesis process uses reverse translation methods for developing the gene DNA sequence. The 20 amino acids are encoded by 4^3^ = 64 nucleotide triplets (codons); some amino acids are encoded by several codons. Genetic code redundancy allows modifying the DNA sequence without changing the protein sequence encoded. The reverse translation method considers different choices from a suite of synonymous codons, taking into account requirements like the absence of regulatory, repetitive, and extraordinarily high GC content subsequences and preferences for particular codons by the host organism. The brute-force approach to reverse translation, screening all possible alternative sequences, rapidly becomes impractical, because the number of such sequences grows exponentially with length of the sequence. Thus, some empirical rules (heuristics) are used in all 37 reported computer programs [10].

In the present paper, we describe a computer program that designs the whole artificial gene synthesis process, developing the optimal nucleotide sequence encoding a given peptide for a given host organism and determining the best long DNA assembly protocol. These tasks are closely connected: synonymous codon substitutions may lead to changes in the optimal assembly protocol, and lack of a simple assembly protocol may be addressed by changing the nucleotide sequence. We also describe an assembly method, where the fragments are designed for carrying out the reaction at a decreased temperature.

Existing programs for gene synthesis that automatically design oligonucleotides are developed mainly for the PCA method [11–15]. An exception is TmPrime [16], which supports the LCR method but requires synthesizing oligonucleotides that fully cover both the coding and the noncoding strands. The approach proposed in this paper allows LCR assembly with gaps in the noncoding strand, which leads to significant reduction in the cost of gene synthesis.

The calculation is a single multicriteria optimization task. The algorithm output is a set of DNA fragments that are short enough to be chemically synthesized and a protocol for their assembly. The melting temperatures of the overlapping regions are slightly different for each pair. If the reaction cannot be performed in one mixture, the possibility of performing the reactions in separate tubes and then joining the results is considered.

## 2. Materials and Methods

The method of producing long DNA molecule encoding a given peptide for a given host organism from shorter fragments synthesized chemically is called the synthesis protocol here. The protocol includes the sequences of shorter fragments and a method for assembling them. A single mixture synthesis is called the base protocol; a multitube synthesis is called a complex protocol. If a given DNA molecule cannot be created correctly by a base protocol (one-tube reaction), for example, because the fragments fold in an incorrect way, a complex protocol is considered (reaction in separate tubes).

### 2.1. DNA Assembly Method

**Figure 1:**
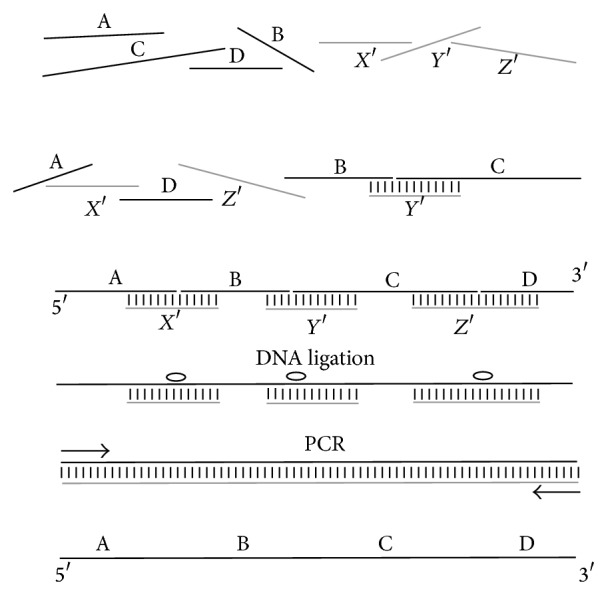
Long DNA base synthesis protocol. Shorter fragments are synthesized chemically; then during hybridization with a slowly decreasing temperature, they form the proper molecule. Next, ligation joins the parts into a longer molecule. Finally, PCR with specific primers amplifies the correct DNA strands. If a correct molecule cannot be created because the fragments fold in an abnormal way, the reaction is performed in separate tubes (complex protocol).

The base synthesis protocol is shown in Figure 1; it is essentially a modified assembly by ligation [8]. Shorter fragments are synthesized chemically and then, by hybridization and ligation, the longer molecule is formed. Finally, PCR with specific starters (primers) is used to amplify the correct DNA strands. The mixture consists of fragments of the desired DNA strand, called* main chains* (the molecules labeled A, B, C, and D in Figure 1 are the main chains), and the* helper chains*—complementary to the two adjacent fragments—are used for joining (molecules* X*,* Y*, and* Z* in Figure 1).

In the assembly protocol presented, the temperature is changed slowly from a temperature high enough to unfold all the fragments to a temperature where all fragments will hybridize. The temperature management thus differs from other assembly protocols, where the folding temperature is similar for all fragments. The change helps to form the correct molecule, because fragments fold in the desired order. The different-temperatures-for-different-fragments feature requires more advanced fragment sequence design. The number of set of fragments considered in synthesizing a given DNA molecule is much bigger, leading to an increase in algorithm time complexity.

Of course, there is no guarantee that every set of fragments will assemble in the desired order. We examine every possible pair of strands from the solution—main and helper chains—including pairs consisting of two identical molecules (fragment with itself). These examinations use DNA secondary structure prediction algorithms, which predict how the strands will fold and what temperature is needed to unfold them. The results, ordered by descending temperatures, define the sequence of strand foldings. This order depends strongly on the sequences of the fragments.

It is desirable that, each time, the fragments in solution are either correctly folded or completely unfolded. A correctly folded fragment is created by a pair that folds into a coherent double-stranded structure with dangling single-stranded ends. If the pair contains a strand that is already folded, then the double-stranded structure is extended either by main chain or by the helping chain. Ultimately, the whole long DNA strand is composed and can be subjected to further treatment, such as completing bonds between the chains by DNA ligase, as shown in Figure 1.

Computer simulations for the base protocol with random sequences of length 60 to 80 nt have shown that about 1% of possible mixtures of three strands (two main chains and one helper chain) lead to correct long molecule creation. In 10000 simulations, the main chains were calculated by drawing, with uniform distribution, the position on the random target sequence, which divided it into two subsequences of length 20 to 40 nt. The helping chain was complementary to a region near this position. Most mixtures were unsuitable for synthesis by the base protocol due to incorrect folding. Fortunately, the number of good solutions can be increased by extending the base protocol to the complex one.

**Figure 2:**
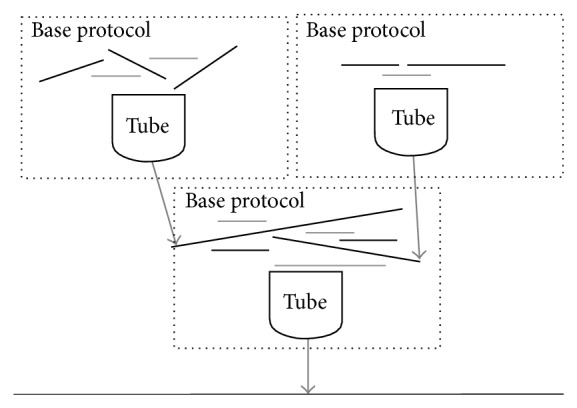
Long DNA complex protocol; multiple tubes are used.

Long strand assembly can be achieved in steps, as shown in Figure 2. In each step, conflicting strands (strands forming improper pairs) are separated into different tube. Then, the base protocol can be used in each tube. Chains formed from the base protocol become the input strands for the next base protocol and the procedure is repeated until the full-length strand is obtained.

Obviously, the complex synthesis protocol is more expensive than the base one. The execution of each step in the laboratory takes about 24 h, multiple probes are needed, and the number of required nucleotides is noticeably greater.

### 2.2. Computer Program Implementation

The application searches the artificial gene synthesis process in the space of possible DNA representations of a given protein and possible DNA assembly protocols described above (base or complex). Each synthesis process is defined by set of DNA sequences (fragment sequences) and a protocol for their assembly (which fragments in which tube). Each synthesis process has a quality measure assigned; this quality is used to optimize in the space of DNA representations and assembly protocols using a combination of an evolutionary algorithm for global optimization and a hill-climbing algorithm to perform fine tuning. The algorithm is depicted in Algorithm 1.

The synthesis process representation is a solution candidate (individual) for the evolutionary algorithm. It is represented by the target DNA strand (in a form of sequence of nucleotides), the collection of positions in this strand describing the places of main chains separation, and another collection of positions, describing the helper chains. Each fragment (main or helper chain) meets the constraints on minimum and maximum strand length (chemical synthesis limitations). Each individual differs in three aspects: nucleotide sequence encoding a given peptide (different codons), main chain fragmentation, and helper chain selection. The number of individuals in a population is an algorithm parameter and can be modified by the user.

The initial set of individuals (initial population) is created as depicted below. The target DNA sequence is created from a given peptide sequence by the chosen codons; the codon encoding a given amino acid is chosen randomly with equal probability. The places of fragment separation are also chosen randomly, with uniform distribution, having regard to the minimum and maximum length of the fragment. The length of a helper chain is random with a uniform distribution between the minimal and maximal length of the fragment. Its sequence is complementary to the sequence around locations of main chain separation.

All individuals were chosen for reproduction. Every parent produces a single mutated child by choosing, with equal probability, one of the following transformations:

- replacement of the random codon (nucleotide triplet) from the sequence,
- increment or decrement of a random position describing the target DNA separation into main chains—one fragment is shortened and the other is extended,
- one randomly chosen helper chain that is shortened or extended or moved left or right.

The fitness of the individual depends on codon frequencies used, the total number of nucleotides used for the artificial gene synthesis, and the number of steps required for assembly (number of required base protocols).

The individual's fitness can be represented by formula (1), where *F*_*C*_, *F*_*N*_, and *F*_*S*_ represent codon frequency component, the sum of the lengths of molecules used for assembly, and the number of base assembly protocols, respectively. The *F*_*C*_ is the root mean square of the differences between required and obtained codon frequencies scaled by weight *w*_*C*_. For codon *j*, *C*_*rj*_ denotes optimal (required) codon frequency, for example, available at [17], *C*_*ij*_ is an individual's codon frequency, *n*_*j*_ is a number of a codon *j*, and *n* = ∑_*j*_ *n*_*j*_ is the number of codons. The calculation of *F*_*N*_ exploits *N*, number of nucleotides used for assembly (sum of all fragments' lengths), *N*_max⁡_ = 2*N*_main_ is the maximum number of nucleotides which can be used, the helping chains have maximum length, *N*_main_ is the length of the target DNA sequence, *N*_min⁡_ = *N*_main_ + (*k* − 1)*L*_min⁡_ is the minimum number of nucleotides which can be used, all helping chains have minimal length *L*_min⁡_ (the algorithm parameter, the property of chemical synthesis), *k* is the number of main chains, and *w*_*N*_ is weight of this component. The calculation of *F*_*S*_ uses weight *w*_*S*_ and *S*—number of base protocols involved in the assembly. Consider

(1) F=11+FC+FN+FS,whereFC=wC·∑jnjCrj−Cij2n,FN=wN·N−Nmin⁡Nmax⁡−Nmin⁡,FS=wS·S−12.

The individual fitness calculations include the determining of optimal assembly protocol. Each pair of fragments, including a pair created from a fragment with itself, is considered in finding the pair's folding order. This order depends on the pair's melting temperature. A modified Zuker algorithm [18] with energy parameters for DNA [19] is used; it is a dynamic programming algorithm to calculate the secondary structure free energy, using thermodynamic data. Our modification allows using the algorithm for pair of strands. Because the folding temperature depends directly on secondary structure free energy, returned by the algorithm, it is used to examine the fragment joining order. If the joining order is improper, the conflicting pair are placed in separate tubes and a base assembly protocol is changed to a complex one.

In every generation, parents and offspring are selected for succession. There are three methods of selection: proportional, ranking, or tournament (*k* = 2). The method is chosen by the user (settings file) as well as the number of generations (stop condition) and some other parameters.

### 2.3. Software Architecture

The application was implemented in client-server, three-layered software architecture, where the presentation layer was deployed on client machine, and the data processing and data storage layer were deployed on server. We developed the software based on a* bioweb* [20] framework; for example, we use C++ and Python and Apache Flex and Java Script programming languages. The C++ language was chosen for algorithm implementation, because it provides the good trade-off between the availability of optimization techniques, portability, and extensibility. The Python was used to connect all server modules; the server is set up on a Django framework. The user interface uses Apache Flex; therefore only web browser is required on client machine.

The tests show that a single individual's fitness calculation time for real-life input needs seconds or even minutes. For example, on a typical 3 GHz CPU core, it takes an average of 90 s to compute a 1000 nt gene synthesis simulation. This was too long for finding the best assembly protocol. Thus, we decided to include additional heuristics to speed up calculations, to use distributed calculations (many computers), and to take advantage of GPU power.

For distributed calculations, we used Common Object Request Broker Architecture (CORBA). An individual's fitness is considered independently, so the calculations can be delegated to different cores, processors, and computers.

The most time-consuming task (99% of the whole application execution time) was the calculation of free energy using the modified Zuker algorithm. The task time-complexity was Θ(*n* ^4^) and with the use of heuristics, this could be simplified to Θ(*n* ^3^ + 30^2^ *n* ^2^). We noticed that a mutation can affect changes only on 1–3 strands in a probe; thus, best efforts were made to minimize the number of such operation calls by storing the results and making them inheritable by mutated individuals. As a result, when the 1000 nt gene synthesis protocol was evaluated, its derivatives were calculated 10 times faster.

To increase the speed of DNA secondary structure prediction calculations, a module was implemented in Compute Unified Device Architecture (CUDA) to take advantage of GPU power. Every subchain is considered independently; thus, it is possible to make these calculations in parallel, and then the results are combined. The calculation speedup was greater for longer strands. For example, a 384 nt sequence was calculated 12 times faster on a Geforce GTX 460 than on a 3 GHz CPU core. The whole application speed increase was also significant; the 1000 nt gene synthesis simulation with GPU support took 40 s, on average, and was 2.5 times faster than with the CPU. These results allowed designing a synthesis of a typical protein in a few hours; however we consider using other algorithms to speed up calculations, for example, using known structures stored in a database [21] and using the information of homologous sequences [22].

## 3. Results

DNASynth application was used for the synthesis of an analogue of the yeast* Saccharomyces cerevisiae Ubp4* protease gene. Protease* Ubp4p* is an enzyme that cleaves ubiquitin from proteins fused to its C-terminus. This gene sequence was described in 1995 (GenBank: CAA89098). The whole gene is 2778 bp; however shorter analogues 276 bp (*Ubp4*′) and 1083 bp (*Ubp4*′′) were designed.

The* Ubp4*′ synthesis involves the 11 fragments designed by the present computer program, depicted in Table 1. These fragments could be mixed in one tube without risk of incorrect connections; a one-step synthesis could be used. An evolutionary algorithm with 200 individuals in the population, 466 generations, and tournament selection found this assembly protocol in 100 h on a PC with z 3 GHz CPU and a nVidia Geforce GTX 460 GPU.

The Ubp4′1, Ubp4′3, Ubp4′5, Ubp4′7, Ubp4′9, and Ubp4′11 fragments had to be phosphorylated. We recommended using commercially phosphorylated primers. Then, 20 pmol of each phosphorylated (Ubp4′1, Ubp4′3, Ubp4′5, Ubp4′7, Ubp4′9, and Ubp4′11) and unphosphorylated primer (Ubp4′2, Ubp4′4, Ubp4′6, Ubp4′8, and Ubp4′10) was placed in a tube with ligation buffer in a volume of 50 *μ*L and heated to a temperature of 94°C. Then, the reaction mixture cooled slowly to room temperature over about 1 h. Next, we added 1 *μ*L of ligase and left it for 10 min at 22°C.

The PCR was performed in a 50*μ*L reaction volume with a buffer containing 50 mM KCl, 2 mM MgCl2, 0.02 mM of each dNTP, 75 mM Tris-HCl (pH 8.9), 20 mM (NH4)2SO4, 25 pM of each primer: Ubp4′Rev, Ubp4′For (with additional sequence for restriction enzymes:* Nde*I,* EcoR*I, and* BamH*I), the enzyme Biotools DNA polymerase (Biotools B&M Labs. S.A. or Taq DNA polymerase with standard Taq buffer, New England Biolabs, Inc.), and 1 *μ*L as a template for 23 and 29 cycles using Eppendorf 5330 thermocycler. Each cycle consisted of 40 s at 94°C, 40 s at 47°C, and 1 min at 72°C for 4 cycles and next, each cycle consisted of 40 s at 94°C, 40 s at 52°C, and 1 min at 72°C for 19 or 25 cycles.

**Figure 3:**
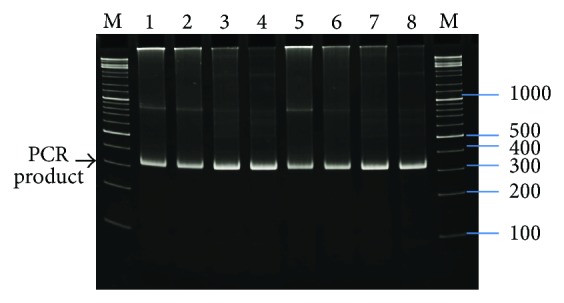
PCR products of UBP4′: M-marker (bp) GeneRuler DNA Ladder Mix (Fermentas Life Sciences), lanes 1–8 UBP4′ gene (276 bp UBP4′ length and 26 bp for restriction enzymes sequence = 302 bp) PCR product: lanes 1, 2: PCR reaction with Biotools DNA polymerase and 23 cycles, lanes 3, 4: PCR reaction with Biotools DNA polymerase and 29 cycles, lanes 5, 6: PCR reaction with Biotools DNA polymerase and 29 cycles, and lanes 7, 8: PCR reaction with Taq DNA polymerase and 29 cycles.

The temperature of annealing was higher for following cycles because of better specificity of the PCR reaction. The amplified 302 bp long DNA fragment was isolated by 1% agarose gel electrophoresis (Figure 3). It was cloned into the pBlue vector and next into expression pT7RSNHU vector at the* Nde*I and* BamH*I sites. The nucleotide sequences of inserts were verified by automatic sequencing. 20 plasmids were analyzed in this manner. The DNA sequence was assembled correctly in 80% of the sequenced plasmids. Results of the sequencing indicate high efficacy and usefulness of DNASynth for obtaining synthetic genes.

**Figure 4:**
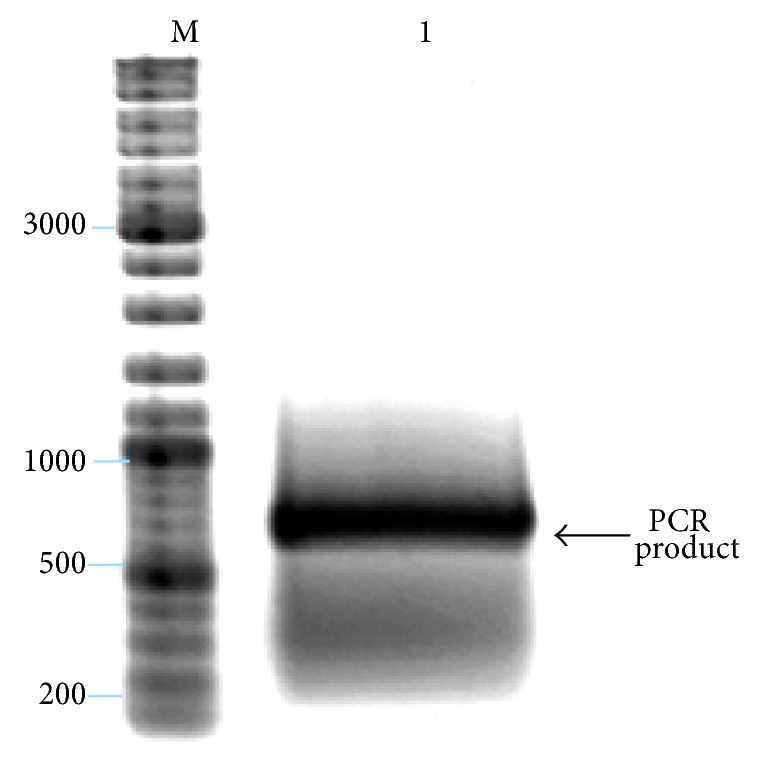
1% agarose gel electrophoresis of PCR products of *UBP4*′′*a*: M-marker (bp) GeneRuler DNA Ladder Mix (Fermentas Life Sciences), lane 1: *UBP4*′′*a* gene (540 bp *UBP4*′′*a* length and 26 bp for restriction enzymes sequence = 566 bp). PCR reaction with Biotools DNA polymerase and 23 cycles.

**Figure 5:**
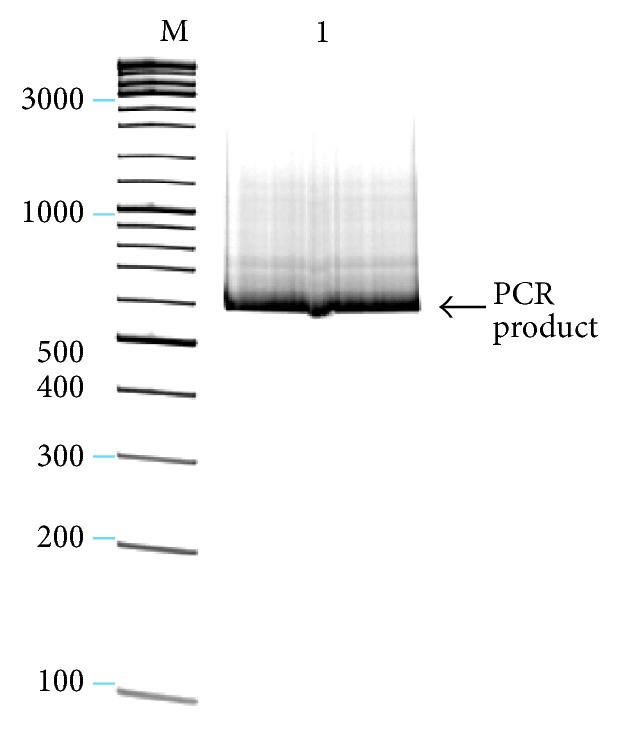
Analysis of the products was performed by 8% acrylamide gel electrophoresis. PCR products of *UBP4*′′*b*: M-marker (bp) GeneRuler DNA Ladder Mix (Fermentas Life Sciences), lane 1: *UBP4*′′*b* gene (543 bp *UBP4*′′*b* length and 26 bp for restriction enzymes sequence = 569 bp). PCR reaction with Biotools DNA polymerase and 23 cycles.

The longer* Ubp4*′′ gene was prepared from 34 fragments. The DNASynth program suggests complex protocol with two tubes,* Ubp4*′′*a* for part of DNA 1–540 indexed and* Ubp4*′′*b* for 541–1083 indexed. The fragments used for* Ubp4*′′*a* synthesis are depicted in Table 2 and the fragments for* Ubp4*′′*b* in Table 3, respectively. The PCR products were obtained as described previously. We obtained PCR products with 566 bp and 569 bp long DNA (Figures 4 and 5).

The* Ubp4*′′ is product of ligation of* Ubp4*′′*a* and* Ubp4*′′*b*. It was cloned into the pBlue 3 vector and next into expression pT7RSNHU vector. The nucleotide sequences of inserts were verified by automatic sequencing. For 20 plasmids analyzed in this manner the DNA sequence was assembled correctly for at least 80% of the sequenced plasmids. The results of sequencing indicate high efficacy and usefulness of DNASynth for obtaining synthetic genes with length exceeding 1000 bp.

## 4. Discussion and Conclusion

The DNA molecules of gene size are routinely created using PCA by research laboratories and specialized companies (Blue Heron Technology, DNA 2.0, GENEART, and others). These molecules assemble the synthetic chromosome or the synthetic genome using isothermal method [23, 24] or other techniques [25, 26]. The presented protocol could speed up these tasks.

The proposed software designs a synthetic gene sequence for the purpose of gene expression. The software would help in obtaining the relevant planned sequence of a specific DNA without the DNA template. It could also be useful at the time when the sequence is received based on, for example, an assay or modification of this gene by* in silico* analysis. The software would be an alternative for chemical gene synthesis.

In relation to the expression of a synthetic gene, an additional modification, namely, substitution of rare codons with frequent codons, in a relevant host has been planned in order to increase the usefulness of the presented algorithm and at the same time to provide an opportunity for obtaining the best possible results in an experimental laboratory.

The computer program presented considers a much larger number of assembly variants for the proposed assembly method to synthesize a given peptide than previous algorithms. It connects the optimal assembly procedure with artificial gene DNA sequence design. The assembly method uses a decreasing mixture temperature to increase the length and reliability of synthesis.

The software and user manual is freely available at http://dnasynth.sourceforge.net/ under GNU LGPL license.

## Figures and Tables

**Algorithm 1:**
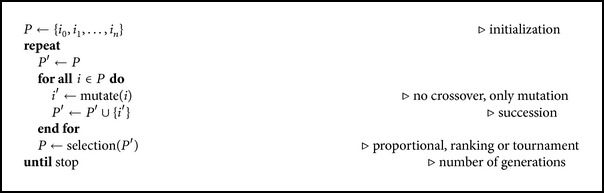
Optimization algorithm used.

**Table 1:** Fragments used to synthesize the *Ubp4*′ gene; Ubp4′1–Ubp4′11 are used in the assembly protocol and Ubp4′For and Ubp4′Rev for PCR.

Name	Sequence
Ubp4′1	A TGTGCTACATGAATTGTATAATACAGTGTATCCTAGGAA
Ubp4′2	AATCTGGGTCAGCTCATGCGTTCCTAGGATACACTGTATT
Ubp4′3	CGCATGAGCTGACCCAGATTTTTCTCGATGACTCCTACGCTA
Ubp4′4	TAGAGTTAATATTGATGTGCTTAGCGTAGGAGTCATCGAGA
Ubp4′5	AGCACATCAATATTAACTCTAAACTAGGCTCTAAGGGCATCTTGG
Ubp4′6	GCACGAGTCGAGCGAAATACTTTGCCAAGATGCCCTTAGAGCCT
Ubp4′7	CAAAGTATTTCGCTCGACTCGTGCACATGATGTATAAAGAGCAAGTA
Ubp4′8	GGGGAAATGGATATTTTCTTAGAACCGTCTACTTGCTCTTTATACATCAT
Ubp4′9	GACGGTTCTAAGAAAATATCCATTTCCCCAATAAAATTTAAATTAGCATGTGGATCCGT
Ubp4′10	GCGGTTTTGAACAACGAGTTTACGGATCCACATGCTAATTTAA
Ubp4′11	AAACTCGTTGTTCAAAACCGCCTCACAACAGGATTGTCAGTAA

Ubp4′For	GGGGGAATTCGATATGTGCTACATGAATTGTATAATAC′
Ubp4′Rev	GGGGGGATCCTTACTGACAATCCTGTTGTGAG

**Table 2:** Fragments used to synthesize the *Ubp4*′′*a* gene; Ubp4′′a1–Ubp4′′a15 are used in the assembly protocol and Ubp4′′aFor and Ubp4′′aRev for PCR.

Ubp4′′a1	T TCGCGGTGGGCCTCGAGAATCTAGGAAATTCCTGCTATATGAACTGCATTATCC
Ubp4′′a2	CAGCTCATGCGTCCCTAAAATACACTGGATAATGCAGTTCATATAGCAGGAATTTCCTAG
Ubp4′′a3	AGTGTATTTTAGGGACGCATGAGCTGACGCAGATCTTCCTTGACGATTCGTATGCGAA
Ubp4′′a4	CCCTAGTTTAGAATTGATATTGATGTGCTTCGCATACGAATCGTCAAGGAAGA
Ubp4′′a5	GCACATCAATATCAATTCTAAACTAGGGTCGAAAGGTATTTTAGCTAAATATTTCGCTCGTCTTGTAC
Ubp4′′a6	TTGGAGCCATCTACCTGTTCCTTATACATCATATGTACAAGACGAGCGAAATATTTAGCT
Ubp4′′a7	ATATGATGTATAAGGAACAGGTAGATGGCTCCAAAAAAATTTCGATTAGCCCAATCAAATTTAAGCT
Ubp4′′a8	AACAGTGAGTTGACGGAACCACAGGCGAGCTTAAATTTGATTGGGCTAATCG
Ubp4′′a9	CGCCTGTGGTTCCGTCAACTCACTGTTTAAAACAGCTTCACAGCAAGATTGTCAGGAATTTTGCC
Ubp4′′a10	GATCTTCATGCAAACCGTCAAGCAAGAATTGGCAAAATTCCTGACAATCTTGCTGTGA
Ubp4′′a11	AATTCTTGCTTGACGGTTTGCATGAAGATCTGAACCAGTGCGGTTCTAACCCC
Ubp4′′a12	CCTCTTGGCTCAGTTCTTTCAACGGGGGGTTAGAACCGCACTGGTTCA
Ubp4′′a13	CCGTTGAAAGAACTGAGCCAAGAGGCAGAAGCGAGGCGGGAAAAGCTGAGCCTC
Ubp4′′a14	GTTCCCATTCAATACTACTGGCTATGCGGAGGCTCAGCTTTTCCCGCCTCGC
Ubp4′′a15	CGCATAGCCAGTAGTATTGAATGGGAACGCTTTCTGACCACGGATTTTAGCGTCATTGTG
Ubp4′′a16	AGCGAGAGGCATATTGCCCTTGAAAGAGGTCCACAATGACGCTAAAATCCGTGG
Ubp4′′a17	GACCTCTTTCAAGGGCAATATGCCTCTCGCTTAAAATGCAAAGTATGCAGCCACACTTCA

Ubp4′′aFor	GGGGGAATTCGATATGTTCGCGGTGGGCCTCGAGAATC
Ubp4′′bRev	GGGGGGATCCTTATGAAGTGTGGCTGCATACTTTGCATTTTAAG

**Table 3:** Fragments used to synthesize the *Ubp4*′′*b* gene; Ubp4′′b1–Ubp4′′b17 are used in the assembly protocol and Ubp4′′bFor and Ubp4′′bRev for PCR.

Ubp4′′b1	A CCACATATCAGCCCTTTACGGTACTGTCTATCCCTATACCCAAAAAGAATA
Ubp4′′b2	AGCAATCCTCGATAGTAATGTTATTGCGACTATTCTTTTTGGGTATAGGGATAGACAGT
Ubp4′′b3	GTCGCAATAACATTACTATCGAGGATTGCTTCCGCGAATTCACCAAATGCGAAAATCTCGAGGTAG
Ubp4′′b4	TTCACAGTGCGGACAGAGCCATTGCTCATCTACCTCGAGATTTTCGCATTTGGT
Ubp4′′b5	ATGAGCAATGGCTCTGTCCGCACTGTGAAAAACGTCAACCGTCAACTAAACAGCTGACCATTACC
Ubp4′′b6	TTTTAGATGGACAATGAGGTTCCGCGGCAAGCGGGTAATGGTCAGCTGTTTAGTTGACGGT
Ubp4′′b7	CGCTTGCCGCGGAACCTCATTGTCCATCTAAAACGTTTTGATAACCTGCTTAACAAAAACA
Ubp4′′b8	AGCAGAAATGGATAAATCACGAAGTCATTGTTTTTGTTAAGCAGGTTATCA
Ubp4′′b9	ATGACTTCGTGATTTATCCATTTCTGCTGGATTTAACGCCTTTTTGGGCAAACGACTT
Ubp4′′b10	TTCACCCCCGGCGGGAACACGCCATCAAAGTCGTTTGCCCAAAAAGGCGTTA
Ubp4′′b11	TGATGGCGTGTTCCCGCCGGGGGTGAACGACGATGAATTACCAATCCGAGGTCAGATCCCGCC
Ubp4′′b12	CACGCGACACCATACAATTCATATTTAAATGGCGGGATCTGACCTCGGATTGGTAATT
Ubp4′′b13	ATTTAAATATGAATTGTATGGTGTCGCGTGCCATTTCGGGACACTGTATGGCGGCCAC
Ubp4′′b14	TTAAACCTTTTTTCACATAAGCCGTATAGTGGCCGCCATACAGTGTCCC
Ubp4′′b15	TATACGGCTTATGTGAAAAAAGGTTTAAAAAAGGGATGGCTTTATTTTGATGA
Ubp4′′b16	GCGTCGGCTTTGTTTTTAACCGGCTTATACTTGGTATCATCAAAATAAAGCCATCC
Ubp4′′b17	TACCAAGTATAAGCCGGTTAAAAACAAAGCCGACGCAATTAATAGCAATGCATATGTTCTGTTCTAT

Ubp4′′bFor	GGGGGGATCCACCACATATCAGCCCTTTACGGTAC
Ubp4′′bRev	GGGGTCTAGATTAATGGTGATGGTGATGGTGATAGAACAGAACATATGCATTGCTATTAATTGCG
